# 11β-Hydroxysteroid Dehydrogenases and Hypertension in the Metabolic Syndrome

**DOI:** 10.1007/s11906-017-0797-z

**Published:** 2017-11-14

**Authors:** Matthew A. Bailey

**Affiliations:** The British Heart Foundation Centre for Cardiovascular Science, The University of Edinburgh, 47 Little France Crescent, Edinburgh, EH16 4TJ UK

**Keywords:** Metabolic syndrome, 11β-Hydroxysteroid dehydrogenases, Hypertension, Glucocorticoid excess, Salt retention, Salt-sensitivity, Aldosterone, Cortisol

## Abstract

The metabolic syndrome describes a clustering of risk factors—visceral obesity, dyslipidaemia, insulin resistance, and salt-sensitive hypertension—that increases mortality related to cardiovascular disease, type 2 diabetes, cancer, and non-alcoholic fatty liver disease. The prevalence of these concurrent comorbidities is ~ 25–30% worldwide, and metabolic syndrome therefore presents a significant global public health burden. Evidence from clinical and preclinical studies indicates that glucocorticoid excess is a key causal feature of metabolic syndrome. This is not increased systemic in circulating cortisol, rather increased bioavailability of active glucocorticoids within tissues. This review examines the role of covert glucocorticoid excess on the hypertension of the metabolic syndrome. Here, the role of the 11β-hydroxysteroid dehydrogenase enzymes, which exert intracrine and paracrine control over glucocorticoid signalling, is examined. 11βHSD1 amplifies glucocorticoid action in cells and contributes to hypertension through direct and indirect effects on the kidney and vasculature. The deactivation of glucocorticoid by 11βHSD2 controls ligand access to glucocorticoid and mineralocorticoid receptors: loss of function promotes salt retention and hypertension. As for hypertension in general, high blood pressure in the metabolic syndrome reflects a complex interaction between multiple systems. The clear association between high dietary salt, glucocorticoid production, and metabolic disorders has major relevance for human health and warrants systematic evaluation.

## Introduction

The metabolic syndrome describes a concurrence of interrelated abnormalities, including visceral obesity, dyslipidaemia, insulin resistance, and hypertension. Each of these features independently carries significant cardiovascular risk. In combination, the risk is amplified, and all-cause mortality increases: metabolic syndrome predicts the development of type 2 diabetes, cardiovascular disease, cancer, and non-alcoholic fatty liver disease [1]. Although a single, unifying definition of metabolic syndrome is lacking, the prevalence of these concurrent comorbidities is ~ 25–30% worldwide [2], presenting a significant global public health burden [3].

Metabolic syndrome is more useful as an epidemiologic tool for analysing cardiovascular risk than it is as a clinical entity requiring specialist management above and beyond management of individual components. For example, hypertension is one of the cardinal features of metabolic syndrome, but the origins of high blood pressure are obscure and lost in the complexity of the syndrome. Clearly metabolic syndrome captures a cluster of pathophysiological features that are individually accepted as “pro-hypertensive”: renal dysfunction and sodium retention [4], vascular [5] and microvascular dysfunction [6], activation of the renin-angiotensin-aldosterone system [7], sympathetic overdrive [8], and oxidative stress [9]. These have all been described in metabolic syndrome patients (and in animal models), as they have for uncomplicated hypertension. Indeed, as for uncomplicated hypertension, it is unlikely that any individual component is “causal”, and there is no distinct blood pressure management strategy for metabolic syndrome patients. Lifestyle and nutritional interventions to increase calorific outflow and lower salt intake are advocated, but adherence is poor, and blood pressure control requires early therapeutic intervention [10]. Nevertheless, there are interesting aspects to metabolic syndrome that may offer a route to improve blood pressure control. Glucocorticoids are important regulators of metabolism. Although rare, the systemic glucocorticoid excess of Cushing syndrome displays the same key features as metabolic syndrome [11]. Although circulating cortisol is not elevated in most patients with metabolic syndrome, “glucocorticoid excess” is a complex concept and may instead reflect instead amplification of cellular bioavailability [12•], enhance frequency/amplitude of pulsatile release over the 24-h cycle [13], and/or alter relationship of circadian/ultradian rhythms to external cues [14••].

This review focusses on covert glucocorticoid excess and the role of local glucocorticoid metabolism by the isozymes 11β hydroxysteroid dehydrogenase types 1 and 2 (11βHSD1 and 11βHSD2). 11βHSD1 and 11βHSD2 are products of distinct genes and members of the dehydrogenase/reductase superfamily. Here, the preclinical and clinical data connecting the activity of these enzymes to blood pressure homeostasis is discussed, concluding by addressing the potential therapeutic relevance to the management of patients with the metabolic syndrome.

## 11βHSDs and Glucocorticoid Signalling

**Fig. 1:**
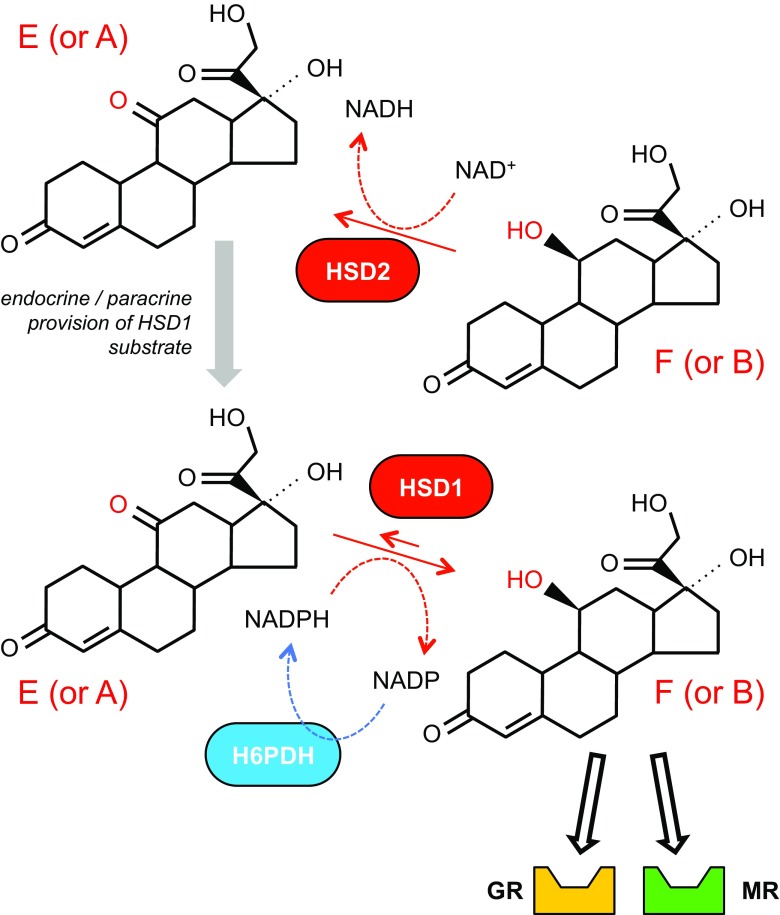
Actions of the 11βHSD enzymes. The bioactivity of glucocorticoid is regulated by enzymatic modification of the C11 side chain. In humans, the reduced 11-hydroxy form cortisol (F) is physiologically active at the mineralocorticoid receptor; the oxidised 11-keto form cortisone (E) is inert. The same is true in rodents for active corticosterone (B) and inactive 11-dehydrocorticosterone (A). Interconversion between the oxidised and reduced forms is catalysed by two 11β-hydroxysteroid dehydrogenase (11βHSD) enzymes. 11βHSD1 operates as an NAPDH-dependent reductase, regenerating active glucocorticoids in target tissues. It is co-expressed in the endoplasmic reticulum with hexose-6-phosphate dehydrogenase (H6PDH), which generates NADPH requisite for reductase activity. 11βHSD2 is a high-affinity NAD^+^-dependent dehydrogenase, inactivating glucocorticoids in vivo. The changes in redox potential that accompany NAD^+^ metabolism may lock MR-cortisol complexes in an inactive state

Plasma concentrations of active glucocorticoid (cortisol in humans; corticosterone in rodents) are determined by the balance between synthesis and clearance, and by the high-affinity binding of glucocorticoids to circulating corticosteroid-binding globulin. Glucocorticoids are synthesised in the *zona fasiculata* of the adrenal cortex in response to ACTH, described as the hypothalamic-pituitary-adrenal axis (HPAA). In peripheral tissues, particularly adipose, liver, skeletal muscle, and kidney, glucocorticoids can be regenerated from inactive 11-keto derivatives (cortisone in humans; 11-dehydrocorticosterone in rodents) by 11βHSD1 (see [12•] for review). Systemic cortisol clearance is primarily mediated by hepatic 5α- and 5β-reductases, with a significant contribution from 11βHSD2 in the distal nephron of the kidney, which converts active glucocorticoids into inactive metabolites (Fig. 1).

The 11βHSD enzymes have conventionally been regarded as regulators of glucocorticoid function at a cell level, but they do exert endocrine influence. Thus, circulating concentration of glucocorticoid is not affected by deletion of 11βHSD2 [15], but the half-life of cortisol is increased in patients with null mutations in the encoding gene, HSD11B2. The effect of 11βHSD1 deletion is also nuanced, appearing as abnormal circadian control of HPAA drive [16]. Micro-dialysis studies suggest that 11βHSDs buffer tissue concentration of glucocorticoid, dissociating this from circulating levels throughout the circadian cycle [17]. Thus, tissue glucocorticoid signalling may be quasi-independent from systemic glucocorticoid status [18]. Mass Spectroscopy imaging is now allowing us to open the black box and peer into tissues: such approaches will significantly advance our understanding of the spatial-temporal regulation of glucocorticoid within tissues and perhaps ultimately cells [19].

Nevertheless, a solely “cellular” view lacks nuance: in humans, 11βHSD1 activity contributes to the postprandial rise in plasma cortisol [20] and in mice 11βHSD2 activity influences the relationship between dietary salt intake and circulating corticosterone [21]. Moreover, gene-targeting strategies in rodents and clinical studies in man clearly demonstrate that these cellular enzymes exert a significant impact on systemic phenotypes, including adiposity and hypertension, discussed below.

## Hypertension in Systemic Glucocorticoid Excess

Iatrogenic or endogenous glucocorticoid excess induces hypertension in humans [22], recapitulated in mice models of chronic corticosterone [23•] and ACTH [24] infusion. Suppression of the endogenous diurnal variation causes a loss of nocturnal blood pressure dipping, even when glucocorticoid stays within the physiological range [25•]. The aetiology and treatment of hypertension in Cushing syndrome has been extensively reviewed [26]. Mechanistically, chronic (5-day) infusion of either ACTH or cortisol into healthy men causes antinatriuresis and volume expansion [27]. Studies in mice show activation of sodium reabsorption in the aldosterone-sensitive distal nephron via ENaC [24] and NCC, the thiazide-sensitive cotransporter [25•]. However, conditional deletion of GR in the distal nephron does not blunt the hypertensive response to chronic dexamethasone [28] (a synthetic glucocorticoid) and long-term glucocorticoid excess causes volume contraction rather than expansion. Here, hypertension is maintained by vasoconstriction due to enhanced sympathetic outflow and increased vasopressin [24]: mice with conditional deletion of GR in the vascular endothelium are partially protected against dexamethasone-hypertension [29]. Nevertheless, it is likely that blood pressure control by the kidney is impaired: the combination of glucocorticoid and sympathetic excess induces salt-sensitive hypertension in otherwise healthy rodents due to epigenetic modification of WNK4 kinase that regulates sodium transport in the distal tubule NCC [30••].

Stable hypertension in ACTH or glucocorticoids excess is often associated with electrolyte abnormalities (e.g. hypokalemia) suggestive of aldosterone excess and in mice ACTH induces increased renal transcription of aldosterone-response genes such as *sgk1* and that encoding αENaC, *scnn1* [31]. As expected, ACTH excess activates gene networks in the adrenal gland that promote steroidogenesis [32], but the effect on circulating aldosterone is transient; the glucocorticoid response is sustained. Thus, GR-mediated pathways are implicated in ACTH-dependent hypertension. MR pathways may come into play if glucocorticoids are sufficiently elevated to breach the 11βHSD2 barrier, as is suggested in human Cushing syndrome [33], or if precursors with mineralocorticoid activity, such as deoxycorticosterone, are elevated to cardiovascular significance [34]. In mice with ACTH excess, both GR and MR antagonism were required to normalise blood pressure [24], and in human ACTH-dependent Cushing, hypertension is often more responsive to mifepristone (RU486) than to MR antagonism [35]. GR antagonism also offers cardiovascular benefits independent of blood pressure control. In a novel rat model of metabolic syndrome, generated by intercross between Dahl-salt-sensitive and Zuker obese rats, RU486 reduced adiposity, 11βHSD1 expression in adipocytes and cardiomyocytes, and reduced cardiac damage without affecting hypertension [36].

## 11βHSD1 and Hypertension

11βHSD1 is highly expressed in the key metabolic tissues of liver, adipose, pancreas, and skeletal muscle. The role of 11βHSD1 in metabolism has been extensively studied from a cellular basis in individual tissues through to impact upon an integrated metabolic system [12•]. A consistent finding in obese humans and rodents is that 11βHSD1 activity in subcutaneous adipose more than doubles (e.g. [37, 38]). Increased adipose 11βHSD1 and consequent intracellular glucocorticoid amplification is similarly reported in patients with metabolic syndrome [39]. Transgenic approaches strongly evidence the relationship between adipose 11βHSD1 and metabolic disease: global knockout mice have a favourable metabolic phenotype, even when obese [40] and adipose-specific deletion protects mice against the metabolic consequences of circulating corticosterone excess [23•]. In contrast, transgenic overexpression of the enzyme in adipocytes markedly enhances cellular glucocorticoid, without changing circulating corticosterone levels, and induces a comprehensive metabolic syndrome phenotype [41]. Importantly, these overexpressing mice have the salt-sensitive hypertension and attenuation of the normal sleep-phase dip [42], characteristic of the blood pressure profile in human metabolic syndrome. In the mice, cellular amplification of corticosterone increased production of angiotensinogen by adipocytes, activating the systemic RAAS [42]. Blood pressure was normalised with an angiotensin receptor blocker, and in metabolic syndrome patients, ARBs offer a safe, effective, and well-tolerated means of blood pressure control [10], with added benefit for other aspects of the syndrome [7].

It is of course challenging to ascribe absolute causality of hypertension in a complex disorder, and several studies show that non-adipose 11βHSD1 activity contributes to blood pressure control. Human genetics studies associate the gain of function rs846910 SNP in the HSD11B1 promotor with blood pressure in non-obese people [43–45]. This SNP associates with type 2 diabetes but not with the metabolic syndrome [46], and such studies offer limited mechanistic insight. However, 11βHSD1 is expressed in systems with a strong influence on blood pressure homeostasis, including vascular smooth muscle cells. It is well-established that glucocorticoids enhance the vasoconstrictor response to catecholamines, yet global 11βHSD1 knockout did not reduce the contractile response to phenylephrine in either the mesenteric artery (resistance) or thoracic aorta [47]. Recent studies show that 11βHSD1 in perivascular fat, amplified in metabolic syndrome [48•], can influence vascular tone: sympathetic over activation increased 11βHSD1 activity and glucocorticoid amplification in perivascular fat, inducing induced endothelial dysfunction in underlying vessels by activation of MR [49••].

The kidney contributes to long-term blood pressure control through the pressure natriuresis, an integrated tubular-vascular response that stabilises extracellular fluid volume [50]. 11βHSD1 is expressed in the renal vasculature and in proximal and distal convoluted tubules, podocytes, macula densa cells, and the interstitial cells of the medulla [51]. Knockdown of 11βHSD1 activity in the rat renal medulla by targeted siRNA delivery decreased the concentration of corticosterone in the urine [52]. This indicates that 11βHSD1 operates as a reductase in vivo despite the absence of H6PDH expression here. Renal medullary upregulation of 11βHSD1 is critical to the hypertensive response to high salt diet in Dahl salt-sensitive rats, and knockdown by the local injection of siRNA is antihypertensive [52]. The molecular mechanisms connecting renal 11βHSD1 activity in the renal medulla to salt-sensitive blood pressure are not resolved, but it is noted that 11βHSD1 null mice are resistant to the hypertension induced by systemic infusion of corticosterone [23•]. It is plausible that 11βHSD1 regulates tubular sodium reabsorption by generating active glucocorticoid, since the stimulatory effects of moderate glucocorticoid excess are well-defined [53]. However, it is unlikely that the enzyme plays a major role in physiological salt balance, since 11βHSD1 knockout mice adapt perfectly well to dietary sodium restriction [54].

The mechanistic relationship between increased 11βHSD1 activity and disorders of metabolism provided a strong driver for development of pharmacological inhibitors. Preclinical studies showed that 11βHSD1 inhibitors lower systemic blood pressure in obese spontaneously hypertensive rats [55]. A blood pressure-lowering effect of a different inhibitor was also observed in mice [56], but this was an off-target benefit, since a similar antihypertensive action was observed in 11βHSD1 knockout mice. In small clinical trials, selective 11βHSD1 inhibitors caused a modest reduction in blood pressure as a secondary endpoint in patients with either type 2 diabetes or the metabolic syndrome [57•]. This was not statistically significant when assessed as a primary endpoint in obese patients [58].

## 11βHSD2 and Hypertension

Mineralocorticoid over-activity is often considered a major factor in the hypertension of glucocorticoid excess. Activation of MR by glucocorticoids is normally restricted by the presence in certain cells of 11βHSD2, which convert MR-active glucocorticoids to MR-inactive metabolites. 11βHSD2 is highly expressed in the kidney, defining the aldosterone-sensitive distal nephron, and here, the activity of the enzyme is certainly important for blood pressure control. Congenital or acquired deficiency in 11βHSD2 causes the syndrome of apparent mineralocorticoid excess (AME; OMIM #218030), presenting with salt retention, potassium wasting, and hypertension [51].

Renal 11βHSD2 activity is regulated by glucocorticoids. It is downregulated following adrenalectomy and restored by corticosterone replacement [59]. Such regulation of 11βHSD2 expression would defend against glucocorticoid-driven sodium retention during periods of physiological glucocorticoid excess. However, recent data from our lab indicate that high doses of dexamethasone actually reduce renal *Hsd11b2* expression [51]. Studies in obese humans also find impaired renal 11βHSD2 activity [60] due to downregulation of gene expression [61]. Renal 11βHSD2 activity is the primary source of 11-dehydrocorticosterone, the substrate for 11βHSD1, and this supply role for peripheral glucocorticoid amplification is metabolically significant [62••]. Thus, metabolic disorders are caught between Scylla and Charybdis: reducing 11βHSD2 activity is metabolically beneficial but increases susceptibility to salt-sensitive hypertension, as demonstrated in the 11βHSD2 knockout rat [15] and discussed below.

In common with other Mendelian disorders, high blood pressure in AME is thought to originate in the kidney [63]. Renal transplant reverses AME in humans [64]; selective deletion of 11βHSD2 in the renal tubule induces key features of AME, including salt-sensitive hypertension [65••]. However, this view is too simplistic. In the global 11βHSD2 knockout mouse, increased vascular tone, reflecting either a defect in endothelial NO production [47] or enhanced sympathetic-induced vasoconstriction [66], maintains hypertension even when sodium balance is restored: activation of the MR target protein ENaC, increases vascular stiffness in obesity [67]. In the CNS, 11βHSD2 is expressed in a subset of neurons in the nucleus of the solitary tract. These neurons are activated by dietary salt restriction. Conditional deletion of 11βHSD2 in the nucleus of the solitary tract does not change blood pressure per se [68] but induces a powerful phenotypic switch from salt-resistant to salt-sensitivity BP so that even modest increases in dietary salt intake cause hypertension [69••]. The switch to salt sensitivity is amplified by an abnormal salt appetite: under free-choice regimens, CNS-knockout mice ingested ~ 3 times more salt than controls [69••].

Mechanistically, null mutations in HSD11B2, or inhibition of the enzyme by glycerrhetinic acid such as found in liquorice [70], would permit activation of MR by cortisol (or corticosterone in rodent models), causing sodium retention [71] due to enhanced reabsorption in the distal nephron via ENaC [66, 72]. Hypertension develops, because HPAA activity is not subject to negative feedback through volume/electrolyte status. However, it is likely that 11βHSD2 in the distal nephron does not act merely as a guardian of renal MR. For example, high salt diet caused moderate glucocorticoid excess in *hsd11b2* heterozygote null mice, and salt-sensitive hypertension was prevented by GR blockade with RU486 rather than by MR blockade with spironolactone [21, 73]. The relationship between 11βHSD2, GR, and MR appears to be more complex than previously thought, at least in the kidney. Here, GR translocation to the cytoplasm is strongly influenced by aldosterone, rather than by physiological levels of corticosterone [74]. It may be that 11βHSD2 also determines the function of GR in “aldosterone-sensitive” cell types. Indeed, studies in a colonic cell line suggest that GR occupancy is a pre-requisite for aldosterone-MR signalling [75]. MR and GR share many of the post-receptor signalling pathways, and a molecular framework for corticosteroid regulation of distal nephron sodium transport—and the role of 11βHSD2 within this framework—is currently being elucidated in our laboratory and in others.

The physiological ramifications of MR/GR interaction are not clear, but it is likely that aldosterone and glucocorticoids normally have mutually reinforcing roles. In a collecting duct cell line, for example, aldosterone activation of MR controls sodium transport during circadian cycles [76]. If aldosterone rises, as seen during salt restriction, activation of GR by aldosterone maximises sodium transport via ENaC. Furthermore, ultradian fluctuations in circulating corticosterone, amplified by renal 11βHSD1 activity, mean that local glucocorticoid may periodically exceed the enzymatic capacity of 11βHSD2. Thus, at key times of the day, or after meals, sodium transport may be physiologically regulated by glucocorticoid. Clearly, this has implications for blood pressure regulation in the metabolic syndrome, where local glucocorticoid excess may underpin enhanced sodium reabsorption in the distal nephron. High-fat feeding to mice recapitulates key features of metabolic syndrome. Impaired sodium excretion and salt-sensitive hypertension reflect activation of furosemide-sensitive NKCC2 [77] and NCC [78], rather than ENaC [79].

## Conclusions

**Fig. 2:**
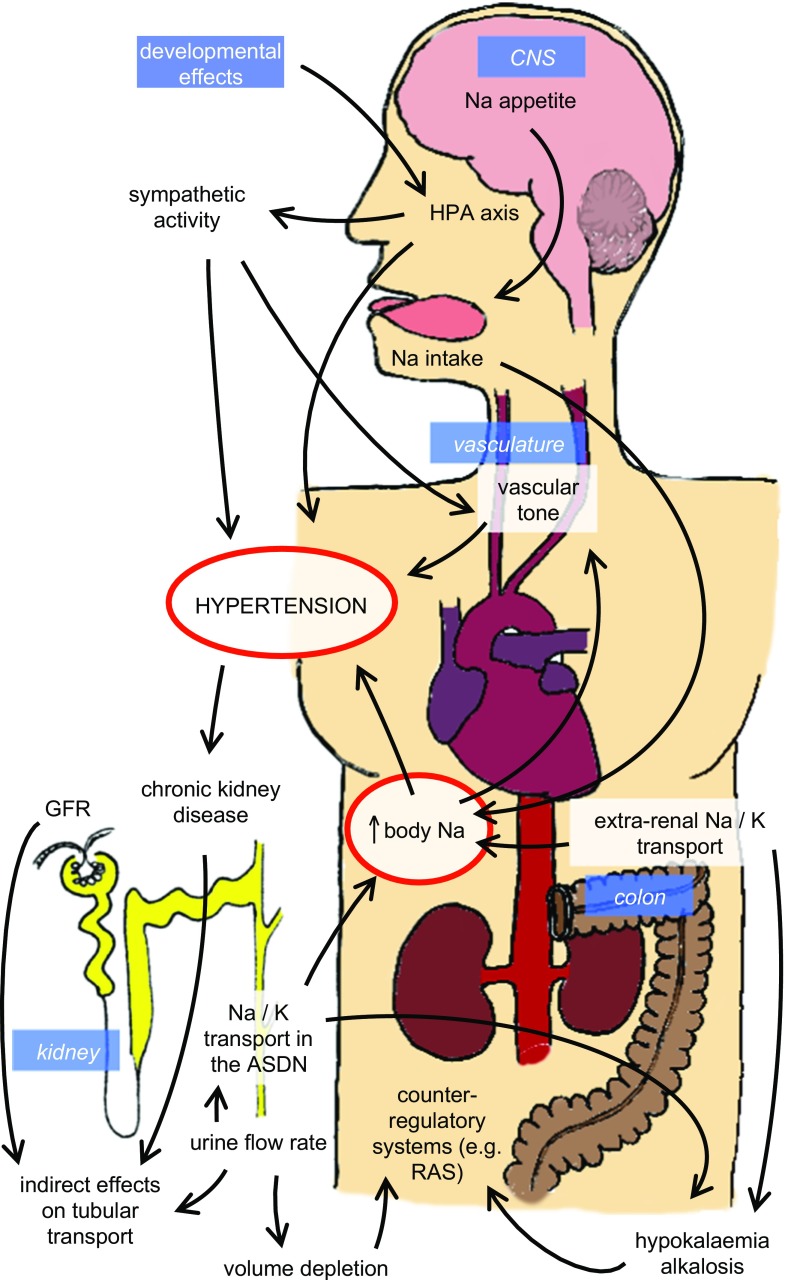
Mechanisms contributing to systemic arterial hypertension in the metabolic syndrome. Hypertension is salt-sensitive and reflects renal, vascular, and central mechanisms. The concept that hypertension is driven by sodium retention arising from renal mineralocorticoid actions is not the whole story, and glucocorticoid receptor blockade is likely to be beneficial. (ASDN aldosterone-sensitive distal nephron)

Conditions associated with increased circulating or intracellular glucocorticoids are common and often associated with hypertension. The metabolic syndrome exemplifies the complexity of glucocorticoid-dependent hypertension: clinical investigation and studies in experimental models demonstrate impairment of all the major homeostatic systems controlling blood pressure (Fig. 2). A unifying factor is that hypertension in the metabolic syndrome is commonly salt-sensitive. This presents a major challenge for clinical management, since salt intake is habitually high, and adherence to salt-restricted diets is notoriously poor [80•]. Moreover, high salt diet itself alters the dynamic regulation of the HPAA. In mice, this manifests as an amplified diurnal peak and enhanced 24-h excretion of corticosterone and metabolites, consistent with enhanced production [21]. In Dahl salt-sensitive rats, high salt diet does not enhance circulating corticosterone but does increase activity of 11βHSD1 in adipocytes [81]. In humans, a direct relationship between salt intake and glucocorticoid production is suggested, also involving peripheral metabolism [82]. Importantly, this relationship between salt intake and glucocorticoid production predicted metabolic syndrome status [83••]. This study was observational, and the association between dietary salt intake, cortisol production, and metabolic disease cannot be regarded as causal. Nevertheless, these relationships have implications for human health and disease and warrant systematic evaluation.
